# Circulatory YKL-40 & NLR: Underestimated Prognostic Indicators in Diffuse Glioma

**DOI:** 10.22088/IJMCM.BUMS.7.2.111

**Published:** 2018-07-29

**Authors:** Puneet Gandhi, Richa Khare, Hanni VasudevGulwani, Sukhpreet Kaur

**Affiliations:** 1 *Department of Research, Bhopal Memorial Hospital and Research Centre, Bhopal, India.*; 2 *Department of Pathology, Bhopal Memorial Hospital and Research Centre, Bhopal, India.*

**Keywords:** Diffuse glioma, YKL-40, neutrophil-lymphocyte ratio, overall survival, prognostic marker, tumor progression

## Abstract

In addition to histopathological parameters, evaluation of associated hematological factors is essential for devising a sensitive prognostic scale in glioma. Increased neutrophil-lymphocyte ratio (NLR), a marker of systemic inflammatory response, has recently been associated with worse outcome in various cancers. Given that glioma progression is characterized by inflammation, aggressive angiogenesis, and invasion, increased levels of systemic human-chitinase-3-like-one protein (YKL-40) have also been linked to poor prognosis. The aim of the present study was to assess the plausible association of YKL-40, NLR, and platelet count with increasing tumor grade, and evaluate their status as independent prognostic factors in terms of overall survival (OS) in treatment naive patients with diffuse glioma. Plasma levels of both biochemical markers in 72 diffuse gliomas, median age 42 years, were compared with 36 controls. Comparison of YKL-40, NLR, and PC with respect to tumor grade was found to be significant for each of the markers (P <0.0001) while an inverse significant correlation was seen for YKL-40 and NLR with OS (r = -0.4619, P <0.0001, and r = -0.5561, P < 0.0001, respectively). NLR was the best performing marker with AUC 0.9417 at 97% specificity. In addition, YKL-40 had a positive correlation with NLR (r = 0.4902, P <0.0001), indicating that expression of both markers was linked to inflammation and tumor progression as they were significantly correlated with tumor grade. Expression of YKL-40 and NLR was independently associated with worse survival (HR 1.0062, P = 0.039, and HR 1.1787, P = 0.0003, respectively), thus establishing their clinical utility as prognosticators for diffuse gliomas.

Gliomas are aggressive and lethal brain cancers (1) with a median survival of 15-18 months (2), and characterized by massive inflammation. This systemic response plays a vital role in cancer progression. Therefore, monitoring inflammation with reference to disease progression is being clinically recognized (3). Chronic inflammation leads to activation of a complex integrated system of mechanisms designed to eliminate the stimulus, repair damaged tissue, and promote wound healing through organized cellular changes that can be measured in blood. The magnitude of activation of this systemic reaction can be measured in terms of circulating white cells, polymorphs, and acute phase proteins (4). In the current decade, blood as a liquid biopsy sample has been recognized as a source of novel biomarkers, and the most practical screening tool. This basis led us to look for circulating inflammatory markers in glioma that could be cost effective and evaluated with minimal invasiveness and higher specificity.

Human chitinase-3-like-protein-1 (CHI3L1, also known as YKL-40), an acute-phase glycoprotein, is elevated in cancers and inflammatory diseases (5) but its clinical application remains restricted. It is secreted by activated macrophages and neutrophils in glioma (6) and is also linked to inflammation (7, 8), angiogenesis (9), cell proliferation, and invasion (10). CHI3L1 is considered to promote Th2-type inflammation as well as enhance tissue remodelling and repair (11). In their study, Low et al. have discussed that an increased *CHI3L1* expression appears to be an important hallmark of inflammation and cancer has been frequently observed in patients with poor prognosis (12).

Similarly, neutrophils are the most abundant white blood cells, and are the first to be recruited to inflammatory sites. The interaction between neutrophils and lymphocytes in response to inflammation is of significance in tumorgenesis (13). In recent studies, neutrophil-lymophocyte ratio (NLR), a haematological marker of systemic inflammation, has been identified as a crucial prognostic biomarker in different cancers (14, 15). An earlier work on glioblastoma by Han et al. demonstrated that pre-treatment NLR was of prognostic significance independent of *O*-methylguanine-DNA methyltransferase (MGMT)  status (16). However, there is a recent contradictory study by Lopes et al. which has stated that there is no correlation between an elevated NLR and worse survival in glioblastoma (17).

Likewise, platelet count (PC) is linked to vessel wall integrity and upon activation; these cells release factors which affect angiogenic activity (18). The present assessment was meant to ascertain the plausible association of YKL-40, NLR, and PC with tumor grade, and evaluate their status as independent prognostic markers in terms of overall survival (OS) in therapy naïve glioma patients.

## Materials and methods

**Patients**

A total of 72 patients admitted for surgery with an initial radiological diagnosis of suspected glioma were considered for this study. Inclusion criteria were- both symptomatic males and females with neurological deficit and no prior chemotherapy or radiotherapy. Glioma grade (II, III & IV) as confirmed by histopathological report, and control subjects without any recent clinical history of inflammation as indicated by their biochemical profile, were taken up for analysis. Exclusion criteria was low grade I glioma (pilocytic).

Variable factors such as age, site of tumor (frontal or non-frontal) and extent of resection in terms of gross total resection (GTR) and subtotal resection (STR) were tabulated from surgical records. The identified biomarkers were compared with baseline values of 36 healthy subjects which served as controls. The study was a part of the project approved by the institutional ethics committee (IEC/21/Res/11) and all subjects provided written informed consent.

All patients were on steroids 24 h prior to surgery. Computed tomography (CT) or magnetic resonance imaging (MRI) was used for initial identification and localization of the tumor. OS time was calculated as the length of time from the start of treatment for glioma till the date of death for a deceased patient, or up to the last follow-up for a surviving patient.

**Sample collection and storage**

Upon obtaining informed consent, 3 ml of peripheral blood was collected from each pre-surgery patient and also from healthy volunteers. Samples were processed at 12 ᵒC, plasma was separated and divided into aliquots, then frozen at -80 °C for further experimentation.

**Blood Count**

The pre-operative total WBC count along with the percentage of neutrophils and lymphocytes were provided by the pathologist to estimate the absolute neutrophils and lymphocyte counts. NLR and PC were then calculated from this full blood count performed before intervention.

**YKL-40 evaluation**

Plasma YKL-40 concentrations (ng/mL) were determined by a sandwich ELISA assay using the commercial kit from R&D Systems, USA (Catalog no. DC3L10). The protocol was executed according to the manufacturer’s instructions. All samples were analyzed in duplicate. YKL-40 levels were measured as absorbance at 450 nm with the correction wavelength set at 540 nm.

**IgE assessment**

Serum IgE levels were recorded from the clinical profile of the patients to confirm that they had no infection at the time of blood sampling. Values were compared within study groups to demarcate that elevated levels of YKL-40 and NLR were due to inflammation on tumor initiation.

**Statistical analysis**

Kruskal-Wallis test was applied to assess the significance of preoperative levels of each biomarker YKL-40, NLR and PC with tumor grade. Mann-Whitney test was performed to compare YKL-40, NLR and PC with OS, when the patients were stratified into 2 groups based on OS of ≤ 15 and >15 months. Spearman’s correlation coefficient was used to calculate the strength of the relationship of markers with tumor grade and OS. With respect to YKL-40, NLR and PC, receiving operating characteristic (ROC) curve analysis was performed to determine the cut-off value of each biomarker for predicting survival with high sensitivity and specificity. Survival curves were made using Kaplan-Meier method, and compared using log**-**rank analysis. Patients who were still alive at last contact were treated as censored events in the analysis. Multivariate Cox regression was performed to calculate hazards ratio (HR) with 95% confidence interval (CI) for each biomarker as a continuous variable. A 2-sided P < 0.05 was taken as statistically significant.

## Results

Patients profile

This is the first cross-sectional study on treatment-naive patients with diffuse glioma (grade II, III & IV) to assess preoperative YKL 40, NLR, and PC as independent prognostic indicators. The reported tumor grade was taken as a base criterion, and the samples were further stratified on the basis of WHO classification, 2016 (19). 23 cases were isocitrate dehydrogenase (IDH)- mutant WHO grade II diffuse astrocytomas, 15 cases were IDH-mutant WHO grade III anaplastic astrocytomas, 4 cases were of anaplastic oligodendroglioma (NOS), and 30 cases of IDH-wild type WHO grade IV glioblastoma. The median age was 42 years, and the cohort had 58 males while 14 were female patients. A median OS of 30, 15 and 6.5 months for grade II-IV was calculated for the cohort (Table 1). Post-operative summary of enrolled subjects presented 32 cases as frontal tumors, with gross total resection achieved only in 14 cases.

**Fig. 1:**
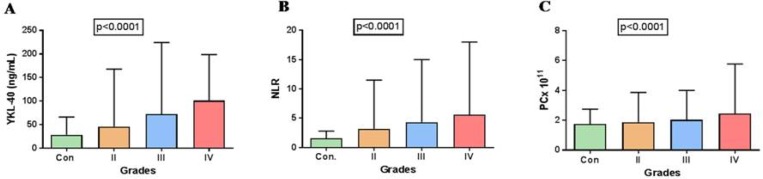
Bar graphs showing the comparison of evaluated blood based markers between tumor grades. A: YKL-40; B: NLR; C: PC. Error bars represent the range of quantified values of each marker

Statistical analysis using Kruskal-Wallis test (non-parametric) for comparison of YKL-40, NLR and PC with respect to tumor-grade was found to be significant for YKL-40 (P < 0.0001), NLR (P < 0.0001), and PC (P <0.0001) (Fig.1) indicating changes in plasma levels with respect to tumor grade when compared with healthy controls.

Mann-Whitney test revealed that all three parameters, YKL-40, NLR, and PC were significantly different in patients with OS of ≤ 15 and >15 months (P <0.0001, P < 0.0001, and P = 0.0002, respectively).

**Correlation**

The correlation of preoperative YKL-40, NLR, and PC with OS was analyzed using Spearman’s rho correlation coefficient. A significant positive correlation of YKL-40 (r = 0.5252, P <0.0001), NLR (r = 0.5041, P<0.0001) and PC (r = 0.3583, P = 0.002) was obtained with respect to tumor grade while a significant inverse correlation of YKL-40 (r = -0.4619, P <0.0001), NLR (r = -0.5561, P <0.0001), and PC (r = -0.4464, P <0.0001) was reached with OS. These statistics indicated that all the three markers were significantly correlated with tumor grade and OS. In addition, YKL-40 had a positive significant correlation with NLR (r =0.4902, P <0.0001), indicating that expression of both markers was linked to inflammation. Since YKL-40 and NLR had a positive correlation with tumor grade, it indicates that as the tumor progresses, the levels of YKL-40 and NLR would increase concomitantly. Hence, both markers can be taken to connote the tumor load of a glioma patient.

**Table 1:** Demographic and evaluated parameters of glioma patients

**Parameters**	**Control** **(n=30)**	**Grade II** **(n=23)**	**Grade III** **(n=19)**	**Grade IV** **(n=30)**
**Age (years)**	25 (21-50)	26 (13-65)	38 (20-75)	51.5 (25-76)
**YKL-40 (ng/ml)**	28.89 (1.119-66.55)	46.08 (14.89-167.987)	72.682(22.448-170.75)	101.15 (21.20-198.77)
**NLR**	1.62 (0.87-2.8)	3.2 (1.23-11.5)	4.33 (2.0-15)	5.59 (3.1-18)
**PC (10** ^11^ **/l)**	1.735 (0.86-2.74)	1.87 (1.17-3.86)	2.02 (1.13-3.63)	2.45 (1.2-5.77)
**OS (months)**	-	40 (16-159)	17 (1-46)	(1-39)

YKL-40: human chitinase-3-like-protein-1; NLR: neutrophil-lymophocyte ratio; PC: platelet count; OS: overall survival. All parameters are presented as median values.

**Table 2:** The cut-off points for biomarkers with their sensitivity and specificity values obtained from glioblastoma circulatory levels

**Biomarker**	**Cut-off Point**	**Sensitivity** **(%)**	**Specificity** **(%)**	**LR**	**AUC of ROC (%)**	**P- value**
**YKL-40(ng/ml)**	> 42.55	80.56	83.33	4.833	87.92	<0.0001****
**NLR**	>2.775	80.56	97.22	29	94.17	<0.0001****
**PC (10** ^11^ **/l)**	>1.790	79.17	58.33	1.9	75.08	<0.0001****

**** highly significant.

**Fig. 2:**
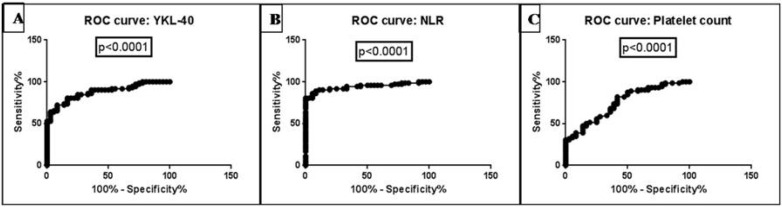
ROC curves of (A) YKL-40, (B) NLR, and (C) PC

**Fig. 3:**
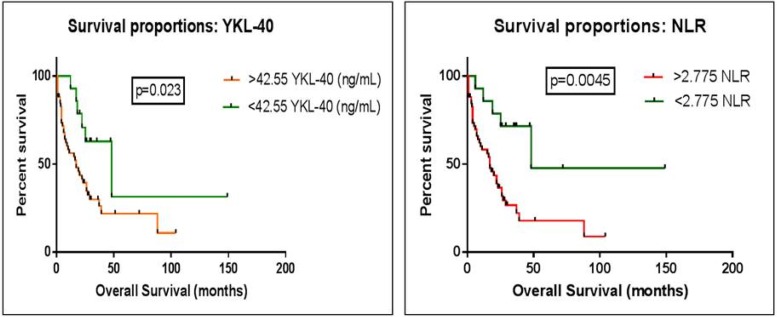
Comparison of survival curves between two defined groups based on ROC cut-offs, to best predict survival. Kaplan-Meier curve for (A) YKL-40 & (B) NLR

**ROC curve analysis**

The AUROC analysis provided an optimal cut-off value for YKL-40, NLR, and PC to differentiate between control and glioma patients. Area under the curve (AUC) for YKL-40, NLR, and PC was 0.8792 (P<0.0001), 0.9417 (P<0.0001), and 0.7508 (P<0.0001), respectively at >80% sensitivity and specificity for all markers (Table 2; Fig. 2 A-C).

**Kaplan-Meier method and log-rank test**

In lieu of the above results, comparison of survival curves between control and glioma patients with optimal cut-off value to best predict survival using log-rank (Mante-Cox) test was undertaken. Patients with a value of YKL-40 exceeding 42.55 were found to differ significantly from those with values <42.55 and had a decreased survival time (17 vs. 48 months, HR 0.3542 with 95% CI: 0.1499 to 0.8365, P = 0.023). Value of NLR exceeding 2.775 was found to differ significantly from those with an NLR quotient of < 2.775, and could even distinguish low grade glioma. A decreased survival time (17 vs. 48 months, HR 0.3542 with 95% CI: 0.1398 to 0.8974, P = 0.0045) (Fig. 3) was recorded in glioma patients. The value of PC did not reach statistical significance (HR 0.375 with 95% CI: 0.1746 to 0.8052, P = 0.0890). Curve analysis depicted that the makers YKL-40 and NLR related significantly with OS, and can thus serve as good prognosticators for glioma.

**Multivariate Cox proportional hazards ratio**

Increase in systemic levels of YKL-40 and NLR was independently associated with worse survival in glioma. When these markers were analyzed as continuous variables, Cox proportional hazards analysis demonstrated a trend towards significantly worse OS for both the prognostic markers YKL-40 and NLR (HR 1.0062, P = 0.039, and HR 1.1787, P = 0.0003, respectively).

We also performed multivariate analysis of the established confounding factors i.e. age, site of tumor, and extent of surgical resection; which showed that higher plasma levels of YKL-40 and NLR were not associated with age (P = 0.174; P = 0.131, respectively), site (P = 0.300; P = 0.4798, respectively), and extent of resection (P = 0.205; P = 0.186, respectively).

## Discussion

The effect of chronic inflammation that develops in the glial tumor microenvironment has far reaching consequences beyond its tumor initiating effect, as it adds on to glioma progression, a stage of the disease that loops back, adding to the intensity of the underlying inflammation.

Experimental studies suggest that inflammation has an important place in the carcinogenesis of high grade gliomas (20, 21) which can be estimated via cells and proteins derived from the peripheral blood. Our test reports indicate the values of NLR to be positively correlated with poor OS (P <0.0001), and tumor grade (P <0.0001), which is in line with our previous study (22), highlighting that NLR score is a good indicator of prognosis and survival in these patients. In agreement our earlier studies carried out only on glioblastoma patients describing NLR to be significantly correlated with OS (23-25) and tumor grading (26). The present study has then provided the first experimental data-based evidence that NLR can be used as an independent prognosticator of all grades of diffuse glioma.

Higher serum levels of inflammatory marker YKL-40 are associated with poor prognosis as ascertained in studies on cancer (27), and also in inflammatory diseases (28-30). Two previous serum-based investigations reported YKL-40 to be associated with tumor grade (31) and OS in high grade glial samples, grade III and IV (32), also indicating potential tumor burden (33). According to Iwamoto et al., increased serum YKL-40 levels were associated with glioma subtypes and poor OS (6), while Bernardi et al. recorded higher serum values among patients with subtotal resection (34). A single study by Gallego et al. on plasma levels of YKL40 suggested that it can be used to differentiate between glioblastoma and non-glial brain tumors (35). The current data also shows plasma YKL-40 to be positively correlated with poor OS (P < 0.0001), NLR (P <0.0001), and progressing tumor grade of patients (P <0.0001), clearly demonstrating tumor load.

In this reference, Schultz et al. have shown that repetitive freezing and thawing of plasma samples up to nine times has no effect on plasma YKL-40 levels (36). They documented YKL-40 in plasma to increase by more than 25% following an inflammatory stimulus, thus making it easily detectable. Since plasma reﬂects the biology of not only tumor cells but also the microenvironment, it becomes a more reliable sample type to evaluate YKL-40 in combination with a panel of other biomarkers for monitoring progress of disease activity.

A significant inverse correlation of PC with OS (P <0.0001) and positive association with tumor grade (P =0.002) was found in test group. In support is the study of 24 glioblastoma patients (37), stating that elevated preoperative platelet counts are associated with significantly shorter survival (38). But our comparison of PC with OS based on AUROC analysis cut-off was non-significant, thereby indicating that PC cannot be taken as a prognostic indicator; which is supported by two earlier negative studies in glioblastoma patients (39, 40).

Our findings demonstrate that values of markers of inflammation, that is YKL-40 and NLR, correlate with tumor grade and OS in therapy-naive diffuse glioma. Comparison of IgE levels within study groups confirmed that elevated values of YKL-40 and NLR were due to tumor burden, establishing the credibility of these circulating markers.

The present investigation clearly delineates that preoperative YKL-40 and NLR can be efficaciously used to follow up diffuse glioma to assess the disease outcome, after adjusting for associated variables of age, tumor site, and extent of resection. The result of this study further identifies the need for validation of these easily accessible, cost-effective, minimally invasive parameters in larger multicentric cohorts; before they can be incorporated into clinical care.
